# Mutation of the *Slt2* ortholog from *Cryphonectria parasitica* results in abnormal cell wall integrity and sectorization with impaired pathogenicity

**DOI:** 10.1038/s41598-017-09383-y

**Published:** 2017-08-22

**Authors:** Kum-Kang So, Yo-Han Ko, Jeesun Chun, Jung-Mi Kim, Dae-Hyuk Kim

**Affiliations:** 1 Department of Molecular Biology, Department of Bioactive Material Sciences, Institute for Molecular Biology and Genetics, Chonbuk National University, Jeonju Chonbuk, 54896 Korea; 2 Department of Bio-Environmental Chemistry, Institute of Life Science and Natural Resources, Wonkwang University, Iksan Chonbuk, 54538 Korea

## Abstract

We assessed the biological function of *CpSlt2*, an ortholog of the cell wall integrity (CWI) MAPK of *Saccharomyces cerevisiae*, in the chestnut blight fungus *Cryphonectria parasitica*. The *CpSlt2*-null mutant exhibited marked changes in colonial growth, near absence of conidiation and aerial hyphae, and abnormal pigmentation. In addition, the *CpSlt2*-null mutant exhibited CWI-related phenotypic defects including hypersensitivity to cell wall-disturbing agents and other stresses. Electron microscopy revealed the presence of abnormal hyphae such as intrahyphal hyphae. In addition, virulence assays indicated that the *CpSlt2* gene plays an important role in fungal pathogenesis. As cultivation of the mutant strains progressed, the majority of the colonies showed sporadic sectorization and mycelia from the sectored area stably maintained the sectored phenotype. Although mycelial growth was partially recovered, the sectored progeny had dramatically impaired virulence, confirming the *CpSlt2* gene has a role in pathogenicity. Compared to a previous mutant of the *CpBck1* gene, a MAPKKK gene in CWI pathway, the *CpSlt2*-null mutant showed similar, although not identical, phenotypic changes and most phenotypic changes were less severe than those of the *CpBck1*-null mutant. These results suggest that the unique sectorization is CWI pathway-specific, though the components in the same CWI pathway have common and specific functions.

## Introduction

At the beginning of the 20th century in North America, the chestnut forests were devastated by *Cryphonectria parasitica* (Murrill) Barr that caused chestnut blight disease (Van Alfen, 1982). Interestingly, single-stranded RNA Cryphonectria Hypovirus 1 (CHV1)-infected strains had characteristic symptoms of viral infection including decreased virulence^1–3^, sporulation, female fertility, pigmentation, laccase production, and oxalate accumulation^4–6^. In virus-infected strains, symptoms include changes in the host transcriptional profiles^7–11^. Since phenotypic changes in virus-infected strains are pleiotropic, a signal transduction pathway during viral symptom development was attributed as a coordinate and specific mechanism of virus-mediated fungal gene regulation. Thus, it has been suggested that in fungi, several signal transduction pathways are perturbed by hypovirus infection^12–18^.

The mitogen-activated protein (MAP) kinases are a family of serine-threonine protein kinases, that comprise the signal transduction pathways of three functionally interlinked cascade kinases: MAPKKK (MAP kinase kinase kinase), MAPKK (MAP kinase kinase), and MAPK (MAP kinase). The MAPK signaling pathways are conserved across a wide variety of organisms from yeast to humans^19, 20^ and are required for numerous processes related to growth, differentiation, and pathogenesis^21, 22^. Five different MAPK pathways regulate mating, filamentous growth, osmotolerance, CWI, and spore wall assembly in *Saccharomyces cerevisiae*. Filamentous fungi are known to have three homologous signaling pathways that regulate the pheromone response, CWI, and the osmoregulation/stress response^21^.

In *C. parasitica*, several genes in MAPK pathways and their downstream effectors are regulated by hypovirus, or are involved in pathogenicity^9, 16, 17, 23–26^. Among these pathways, the CWI pathway has aroused interest because *Cpkk1*, an ortholog of yeast Mkk2 (a CWI MAPKK) was found to be modulated by hypovirus and was important for virulence in the chestnut tree^25, 27^. In addition, recent studies on a mutant of *CpBck1*, an ortholog of yeast Bck1 (a CWI MAPKKK), showed characteristic phenotypic changes related to CWI and virulence^26^. Interestingly, sporadic sectorization, which has not been observed in any other fungi, was observed in the *CpBck1* mutant and sectored phenotypes were stably inherited in the progeny that were successively transferred from sectored mycelia. Thus, in this study, we analyzed the biological function of *CpSlt2*, an ortholog of yeast Slt2, to determine if only *CpBck1*, but not *CpSlt2*, is implicated in sectorization, and if the CWI pathway plays a key role in sectorization.

## Results

### Database description

An analysis of the draft *C. parasitica* genome sequence (http://genome.jgi-psf.org/Crypa1/Crypa1.home.html) using yeast *Slt2* gene identified *CpSlt2*, an ortholog of the yeast *Slt2* gene, which showed high similarity to known fungal *Slt2*-like genes. The corresponding *CpSlt2* gene was PCR amplified and the resulting 4,385-bp PCR amplicon was cloned and sequenced. Based on *in silico* sequence analysis, we performed RT-PCR to obtain a near full-length cDNA clone. The resulting 1,257-bp amplicon was cloned and sequenced. A sequence comparison with the corresponding genomic DNA revealed that the cloned gene consisted of five exons, with four intervening sequences of 89, 99, 86, and 125 bp. The deduced *CpSlt2* protein product (CpSLT2) was shown to consist of 418 amino acids, with a predicted molecular size of 47.4 kDa and a pI of 5.47 (GenBank No. KY620036). The deduced amino acid sequence of CpSLT2 contained all conserved protein kinase subdomains and a canonical TEY sequence found in the phosphorylation site. The sequence environment around the start codon was in good agreement with Kozak’s consensus sequence, in that the −3 position was an A in CAAGATG. However, a typical poly(A) signal (AATAAA) was not found downstream of the stop codon.

Homology searches using the protein product of the *CpSlt2* gene showed that the cloned *CpSlt2* gene exhibited high amino acid identities with other known fungal SLT2 homologs of *Aspergillus nidulans* (85% aa identity), *A. oryzae* (84%), *Claviceps purpurea* (84%), *Colletotrichum lagenarium* (91%), *Fusarium graminearum* (91%), *Magnaporthe oryzae* (91%), *Mycosphaerella graminicola* (83%), and *S. cerevisiae* (62%). In addition, the deduced amino acid sequence of the *CpSlt2* gene harbored a Ser/Thr protein kinase domain found in proteins of the PKc-like superfamily between aa 23 and 314. This domain had high amino acid identities to that of *A. nidulans* (97% aa identity), *A. oryzae* (96%), *C. purpurea* (94%), *C. lagenarium* (97%), *F. graminearum* (98%), *M. oryzae* (98%), *M. graminicola* (93%), and *S. cerevisiae* (72%). A phylogenetic analysis of homologs from other signaling pathways, such as the pheromone responsive pathway (Fus3/Kss1) and the osmoregulation responsive pathway (Hog1), showed that the cloned *CpSlt2* gene belonged to the *Slt2*-related group of MAPKs (Supplementary Fig. S1).

### Construction of the *CpSlt2*-null mutant (TdSLT2)

We examined the biological function of the *CpSlt2* gene using a *CpSlt2*-null mutant, which was obtained through site-directed recombination during integrative transformation. A total of 120 putative transformants were successively transferred to hygromycin B-containing selective medium and 60 stable transformants were obtained. The stable transformants were single-spored, and single-spored transformants were further analyzed to confirm the presence of the expected gene replacement. PCR analysis using two outer (5′distal and 3′distal) gene-specific primers resulted in that two transformants showed a 2.6-kb PCR amplicon, consistent with the expected size of the replaced alleles of the *CpSlt2* gene. Southern blot analysis confirmed the replacement of the wild-type allele with the replaced allele in these two transformants. As shown in Supplementary Fig. S2, the hybridization pattern of *Sac*II-digested genomic DNA from the two putative *CpSlt2*-null mutants using a probe revealed a single hybridizing band at 3.7 kb, which differed from that of the wild type at 2.5 kb, consistent with the expected size of the replaced allele. In addition, the hybridizing band at 3.7 kb also hybridized to a probe from a 0.3-kb *Bam*HI/*Sal*I hygromycin B resistance (*hph*) gene fragment (data not shown), suggesting that a part of the corresponding 3′ proximal region of the *CpSlt2* gene was replaced with the *hph* gene. Thus, PCR and Southern blot analysis showed that these two transformants (named TdSLT2-42 and −69 for 42th and 69th transformants resulting from deletion of SLT2) contained transforming vectors that had undergone double-crossover recombination at the *CpSlt2* locus, resulting in the replacement of the wild-type allele with the appropriate part of the transforming vector without additional integration.

### Phenotypic changes in the TdSLT2 strain

**Figure 1:**
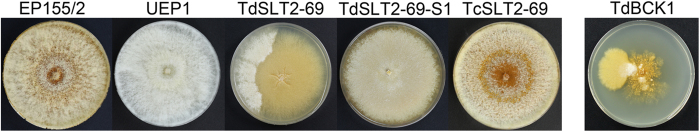
Colony morphology of the *CpSlt2*-null mutant (TdSLT2-69) strain. Strains, as indicated on the panels, are wild-type EP155/2, hypovirulent UEP1, TdSLT2-69, a sectored progeny of TdSLT2-69 (TdSLT2-69-S1), a complemented strain of TdSLT2-69 (TcSLT2-69), and the *CpBck1*-null mutant (TdBCK1) strain. Colonies after 14 d of culture are shown. Note that TdSLT2-69 colonies show the sectored phenotype. The *CpBck1*-null mutant (TdBCK1) (i.e., a mutant strain of MAPKKK in the same CWI pathway) is shown as a comparison.

The *CpSlt2*-null mutants exhibited considerably altered colony morphology (Fig. 1). The mutants had reduced growth rates (less than 60% of that of wild type) and low density mycelia. The *CpSlt2*-null mutants displayed distinctive invasive feeding hyphae at the colony margins that were thinner than that of wild type, a very limited mycelial mat on the surface, and near absence of aerial hyphae and conidia. The pigmentation was observed in the *CpSlt2*-null mutants. The *CpSlt2*-null mutants had pigmentation; however, the pigmentation was not a typical bright yellow color, but rather a diffused pale greyish brown as the culture continued to grow. No differences were observed between the two independent *CpSlt2*-null mutants, TdSLT2-42 and TdSLT2-69. In general, the *CpSlt2*-null mutants showed retarded growth and abnormal morphogenesis. Although the growth defects and morphogenetic alterations were similar, but to a lesser extent, to that of the previously described *CpBck1* mutant (a MAPKKK ortholog in the same CWI pathway), the phenotypic changes were markedly recovered compared to that of the *CpBck1*-null mutant (Fig. 1).

In addition, as culture continued, the *CpSlt2*-null mutants began to display sectorization, as shown by abrupt vigorous growth of shortened aerial hyphae. Compared to the *CpBck1*-null mutant^26^, the occurrence of sectorization per plate was low (three to four out of ten cultured plates) and the number of distinctive origins of sectorization per plate was reduced to one or two. When the *CpSlt2*-null mutants were cultured on minimal medium at a temperature of 20 °C instead of 25 °C, no differences in sectorization were observed (data not shown). Once sectored, the sectored progeny maintained their sector characteristics during successive subcultures and did not revert back to wild type or the parental *CpSlt2*-null mutant.

The phenotypic changes of the *CpSlt2*-null mutant and its sectored progeny in liquid culture were compared. The primary inoculum of 0.2 g of freshly harvested mycelial mat was homogenized in 50 ml sterile medium, and the resulting slurry was used to inoculate 150 ml liquid medium^16^. The parental *CpSlt2*-null mutant showed significantly reduced mycelial mass with abnormal, i.e., pale-greyish brown, pigmentation compared with that of the wild type. The sectored progenies of the *CpSlt2*-null mutant had a mycelial mass similar to that of the wild type, but their pigmentation was not fully recovered, indicating that the phenotypic changes of the mutants were not affected by the culture medium.

Parental *CpSlt2*-null mutants and a sectored progeny of a *CpSlt2*-null mutant (named TdSLT2–69-S1 for a sectored progeny of TdSLT2–69) underwent complementation via *in trans* transformation of corresponding mutants with a wild-type *CpSlt2* gene. PCR amplification of the wild-type allele of *CpSlt2* confirmed that all of the complemented transformants acquired a wild-type allele of *CpSlt2*. The *in trans* complemented strains (named TcSLT2-69 for a transformant resulting from complementation of SLT2) recovered the characteristics of wild type such as conidiation and pigmentation (Fig. 1), indicating that the mutant phenotypes were caused by *CpSlt2* gene.

**Figure 2:**
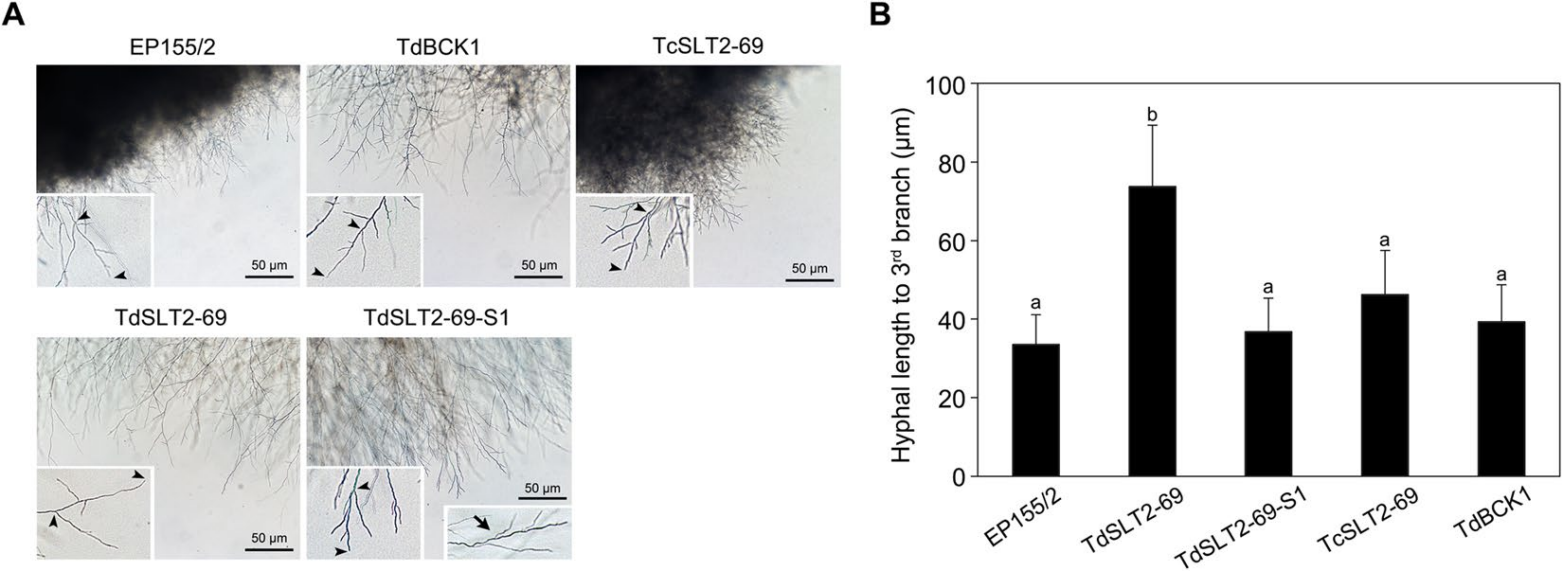
Characteristics of the apical hyphal growth units in the *CpSlt2*-null mutant strain. (**A**) Microscopic observation of apical hyphae of the *CpSlt2*-null mutant strain. Strains, indicated on the panels, are wild-type EP155/2, TdSLT2-69, a sectored progeny of TdSLT2-69 (TdSLT2-69-S1), a complemented strain of TdSLT2-69 (TcSLT2-69), and the *CpBck1*-null mutant (TdBCK1) strain. Note that arrowheads in the enlarged inlet represent examples of the apical hyphal growth unit and the sectored strains are moderately more geniculate or inflective as indicated by the arrow in the enlarged inlet at the bottom right. Black bars represent 50 μm. (**B**) Apical growth units were measured by the hyphal length from the apex to the third branching point. At least 40 individual hyphae were observed for each strain and each experiment was repeated three times. Data followed by the same letters above the bars are not significantly different between treatments, according to the Tukey HSD test at *p* = 0.01.

Microscopic observation of the *CpSlt2*-null mutant showed that the hyphal growth unit, as determined by the length of the hyphae from the apex to the third hyphal tip was significantly larger than that of wild type (Fig. 2). Interestingly, sectored progenies from *CpSlt2*-null and *CpBck1*-null mutant strains showed no difference in hyphal growth unit compared with wild type. In addition, individual hyphae at the apical region of sectored progeny from *CpSlt2*-null mutants were moderately more geniculate and/or showed abrupt changes in curvatures compared with wild type or parental *CpSlt2*-null mutants, which had straight hyphae (Fig. 2A).

**Figure 3:**
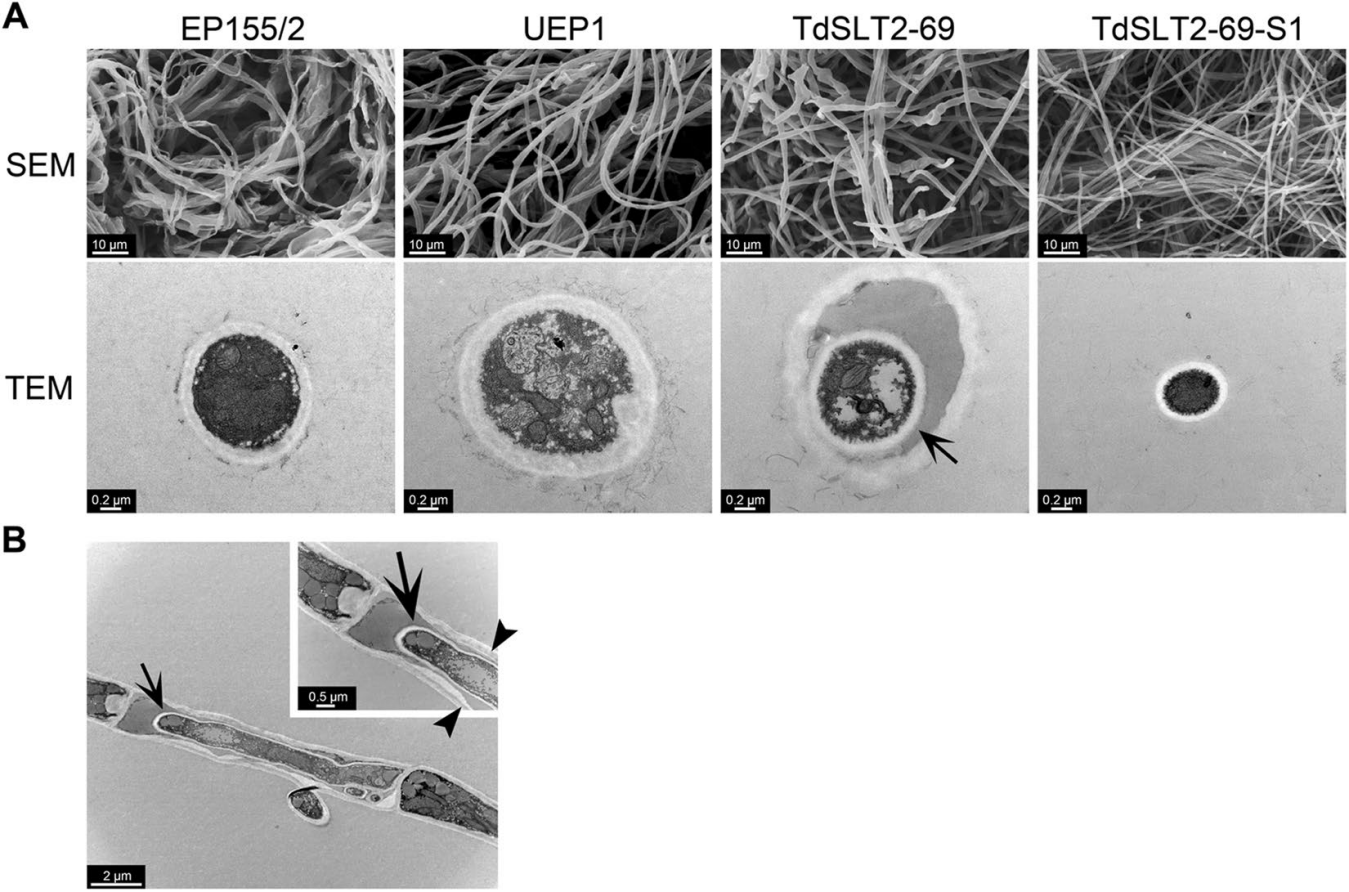
Ultrastructural characteristics of the *CpSlt2*-null mutant strain (TdSLT2-69). (**A**) Scanning electron micrographs and transmission electron micrographs of mycelia. Typical extended filamentous hyphae are observed in the wild-type EP155/2, UEP1, TdSLT2-69, and TdSLT2-69-S1 strains. More dense and thinner individual hyphae with coalesced mycelia are observed in the sectored TdSLT2-69 progeny, TdSLT2-69-S1. Transmission electron micrographs of intrahyphal hyphae of TdSLT2-69 are indicated with an arrow. (**B**) Hyphae with less electron-dense material are noted in the outer cells of intrahyphal hyphae and arrowheads in the enlarged inlet indicate moderately hypertrophied invaginated walls.

We conducted electron microscopy to examine ultrastructural changes in the *CpSlt2*-null mutant (Fig. 3). Scanning electron microscopy (SEM) revealed that the *CpSlt2*-null mutants had slightly swollen but mostly normal hyphae elongation, which differed dramatically from that of *CpBck1*-null mutants. In contrast to the *CpBck1*-null mutant, the *CpSlt2*-null mutants had a lot less frequency of hypertrophied globose or bulbous cells in the hyphae. However, transmission electron microscopy (TEM) showed that the *CpSlt2*-null mutants displayed characteristic proliferation of new intracellular hyphae, termed intrahyphal hyphae or cell-within-a-cell^28–32^. The sectored progeny of the *CpSlt2*-null mutants had normal elongated hyphae but a more compact mycelial distribution with numerous aggregations of individual hyphae, which can be attributed to geniculate rather than normal straight hyphae. However, no distinctive ultrastructural changes were observed in the sectored progeny.

### Responses to environmental changes and cellular stress

Since CWI increases multi-stress tolerance by fortifying the cell wall and repairing cell wall damage after stress^33–35^, we examined *CpSlt2*-null mutant responses to various external stimuli.

We assessed temperature sensitivity at 20 and 25 °C. As shown in Supplementary Fig. S3, compared with wild type, the *CpSlt2*-null mutant exhibited retarded growth, as expected. However, no discernable differences were found in the *CpSlt2*-null mutant in response to cold temperature at 20 °C. Specifically, there were no differences in the frequency of sectorization i.e., similar ratio of sectorization occurred at two different temperatures.

**Figure 4:**
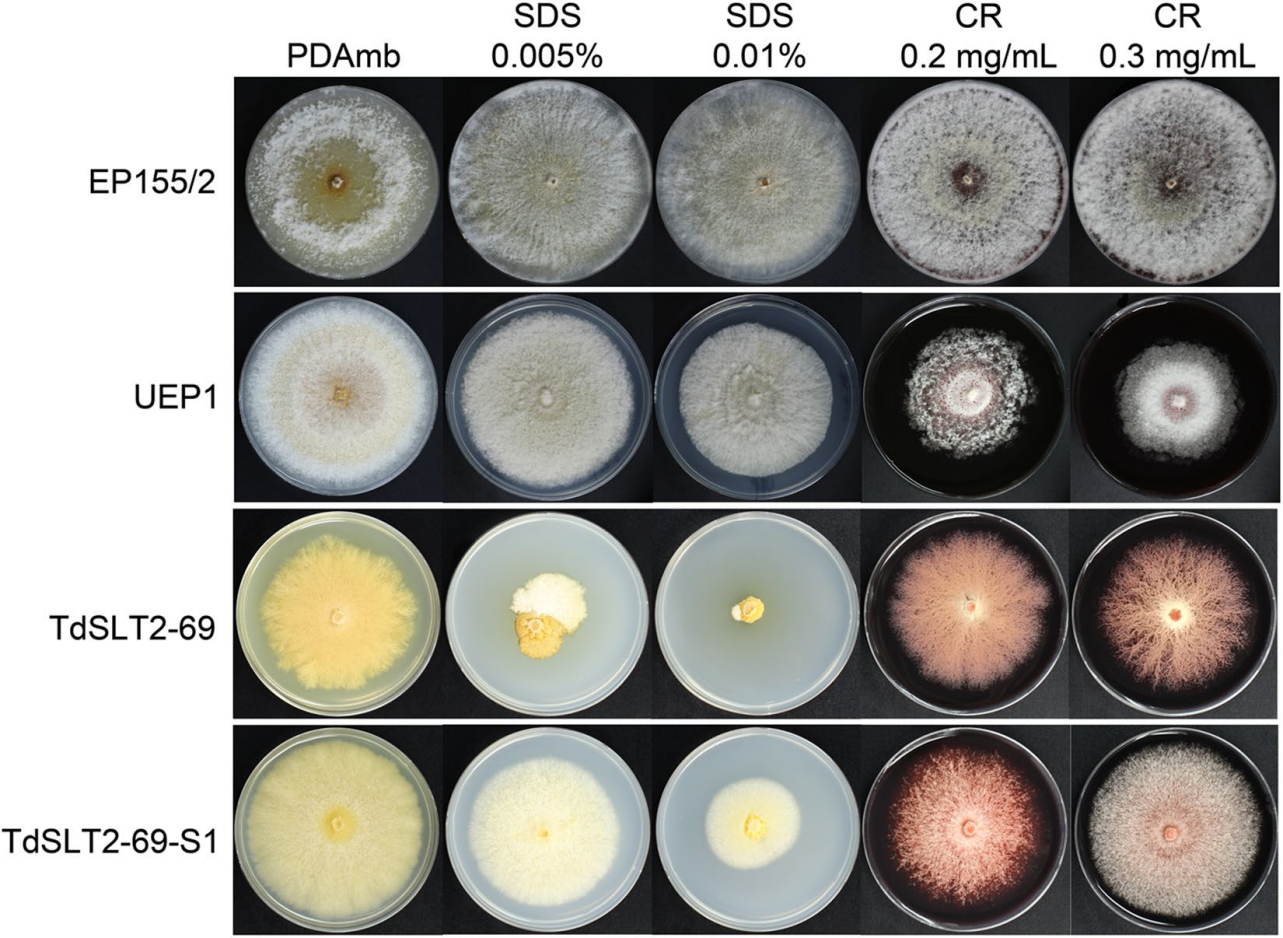
Effect of various cell wall-perturbing agents on fungal growth. Colony morphologies on PDAmb supplemented with corresponding agents are shown after 14 d of cultivation. Strains, indicated at the left, are wild-type EP155/2, UEP1, TdSLT2-69, and the sectored TdSLT2-69 progeny, TdSLT2-69-S1. CR and SDS represent Congo red and sodium dodecyl sulfate, respectively. The radial growth of *CpSlt2* mutants with the same background, such as TdSLT2-69 and TdSLT2-69-S1, showed more severe growth defects on plates containing cell wall-disturbing agents.

Next, we examined wild type and *CpSlt2*-null mutant responses to the cell wall-perturbing agents Congo red (CR) and sodium dodecyl sulfate (SDS). These cell wall-perturbing agents are known to bind to cell wall components and thus interfere with cell wall composition. As shown in Fig. 4, strains with the same *CpSlt2* mutant background, including the *CpSlt2*-null mutant and its sectored progeny, had more severe growth defects when cultured on plates containing cell wall-perturbing agents. This cell wall impairment, as evidenced by increased sensitivity to cell wall-perturbing agents, is consistent with the function of the cloned *CpSlt2* gene as the MAPK component of the CWI signaling pathway.

**Figure 5:**
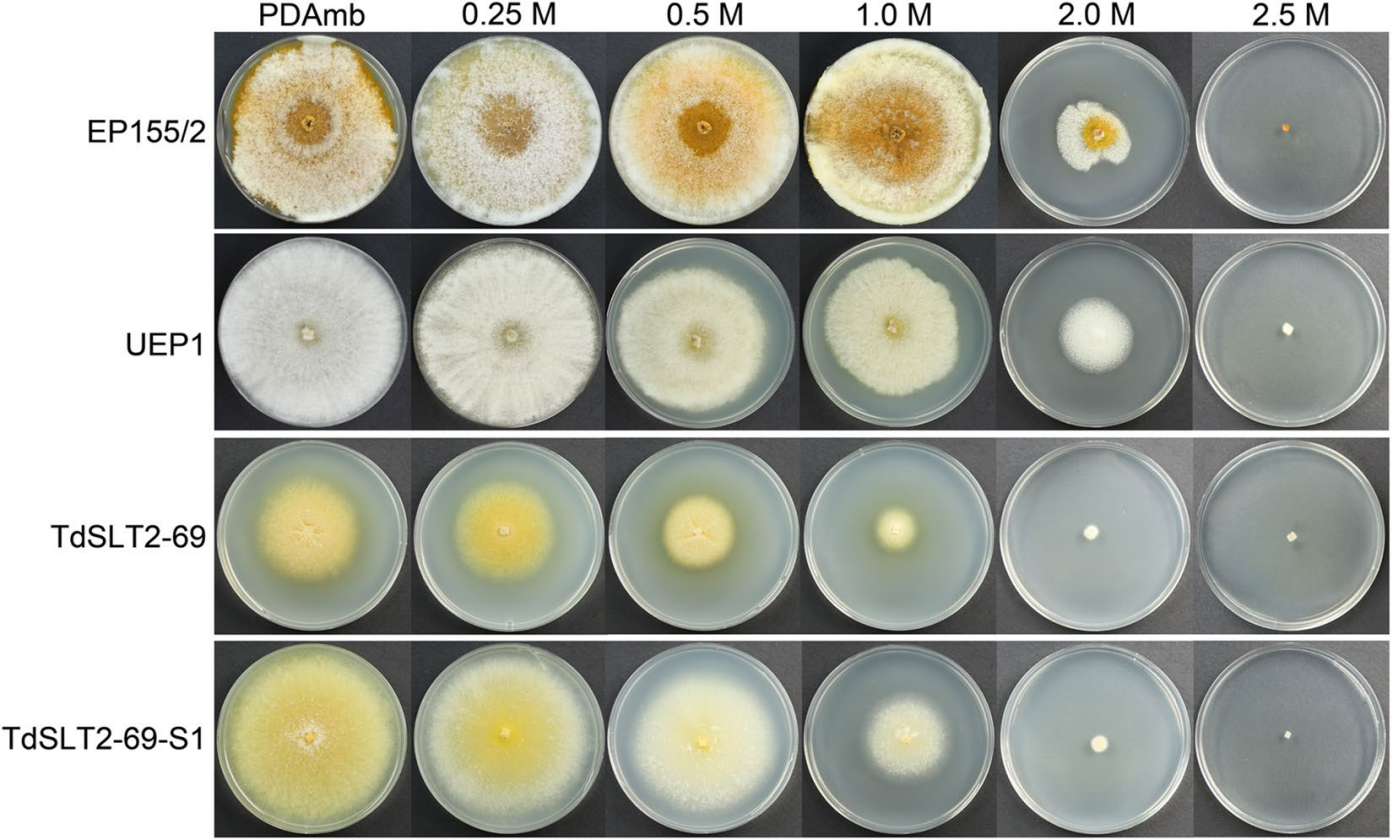
Effect of supplementation with various concentrations of an osmotic stabilizer on fungal growth. Colony morphologies on PDAmb supplemented with corresponding concentrations of sorbitol are shown after 10 d of cultivation. Strains are wild-type EP155/2, UEP1, TdSLT2-69, and the sectored TdSLT2-69 progeny, TdSLT2-69-S1. Note that colonies with the same *CpSlt2* mutant background, such as TdSLT2-69 and TdSLT2-69-S1, showed more severe growth defects when cultivated on plates containing 1.0 M sorbitol. On the other hand, wild type and its isogenic hypovirulent UEP1 strains cultivated in the presence and absence of 1.0 M sorbitol were not significantly different.

We also analyzed responses to osmotic stress. As shown in Fig. 5, supplementation with low and medium concentrations of osmotic stabilizer (<1.0 M sorbitol) did not restore growth of the *CpSlt2*-null mutant. Interestingly, under high osmotic stress conditions (1.0 and 2.0 M sorbitol), a severe growth defect was observed in the *CpSlt2*-null mutant and its sectored progeny, suggesting that the CWI pathway may mediate multi-stress responses via crosstalk with other stress response signaling pathways such as the high osmotic glycerol (HOG) pathway^36, 37^.

We analyzed responses to reactive oxygen species (ROS) stress by growing the strains on PDAmb supplemented with appropriate concentrations of menadione. Compared with wild type, the *CpSlt2*-null mutant and its sectored progeny showed retarded growth in a dose-dependent manner up to 100 μM menadione. These results clearly indicate that the *CpSlt2* gene plays an important role in ROS stress response (Supplementary Fig. S4).

**Figure 6:**
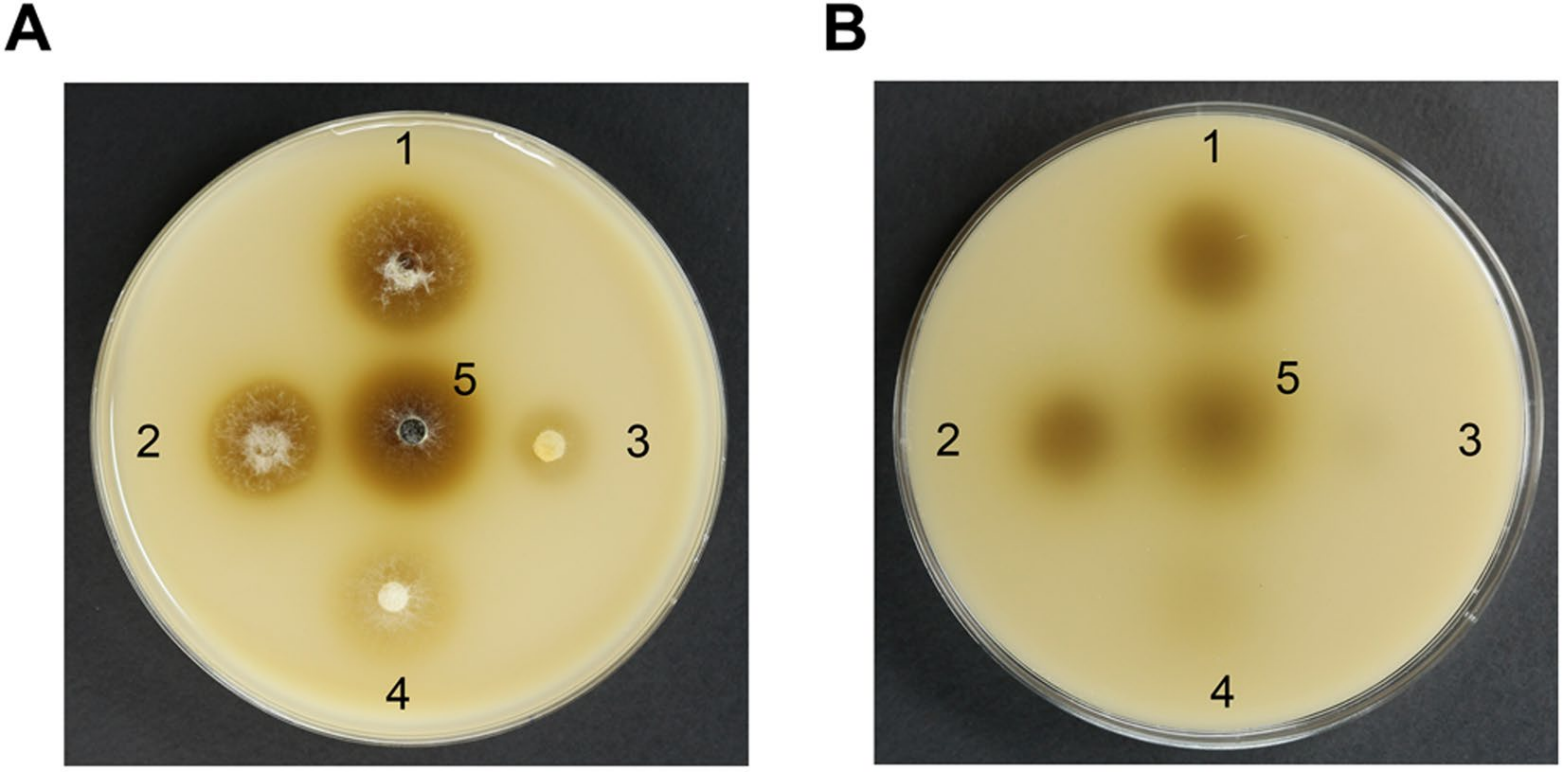
Bavendamm’s assay. Colonies were grown for 3 d on tannic acid-containing medium, as described previously^6^. The level of brown coloration correlates with the polyphenol oxidase activity of each strain. Top (**A**) and bottom (**B**) views of Bavendamm’s plates are shown. The numbers 1, 2, 3, 4, and 5 indicate wild-type EP155/2, UEP1, TdSLT2-69, the sectored TdSLT2-69 progeny, TdSLT2-69-S1, and the complemented TcSLT2-69 strains, respectively.

We examined phenoloxidase activity using the Bavendamm reaction. As shown in Fig. 6, the area of dark brown coloring, which is indicative of phenoloxidase activity, was greatly reduced by the *CpSlt2*-null mutant and its sectored progeny compared with the wild-type strain. The dark brown area was even smaller than that of the hypovirulent UEP1 strain. These results indicate that the production of phenoloxidase was greatly hampered by mutation of the *CpSlt2* gene, regardless of the mycelial growth defects, suggesting that phenoloxidase activity may play a role in maintaining fungal CWI or, aside from CWI, this signaling pathway modulate phenoloxidase production resulting in new aspects of phenoloxidase-mediated biological function of the CWI pathway.

### Virulence assay

**Figure 7:**
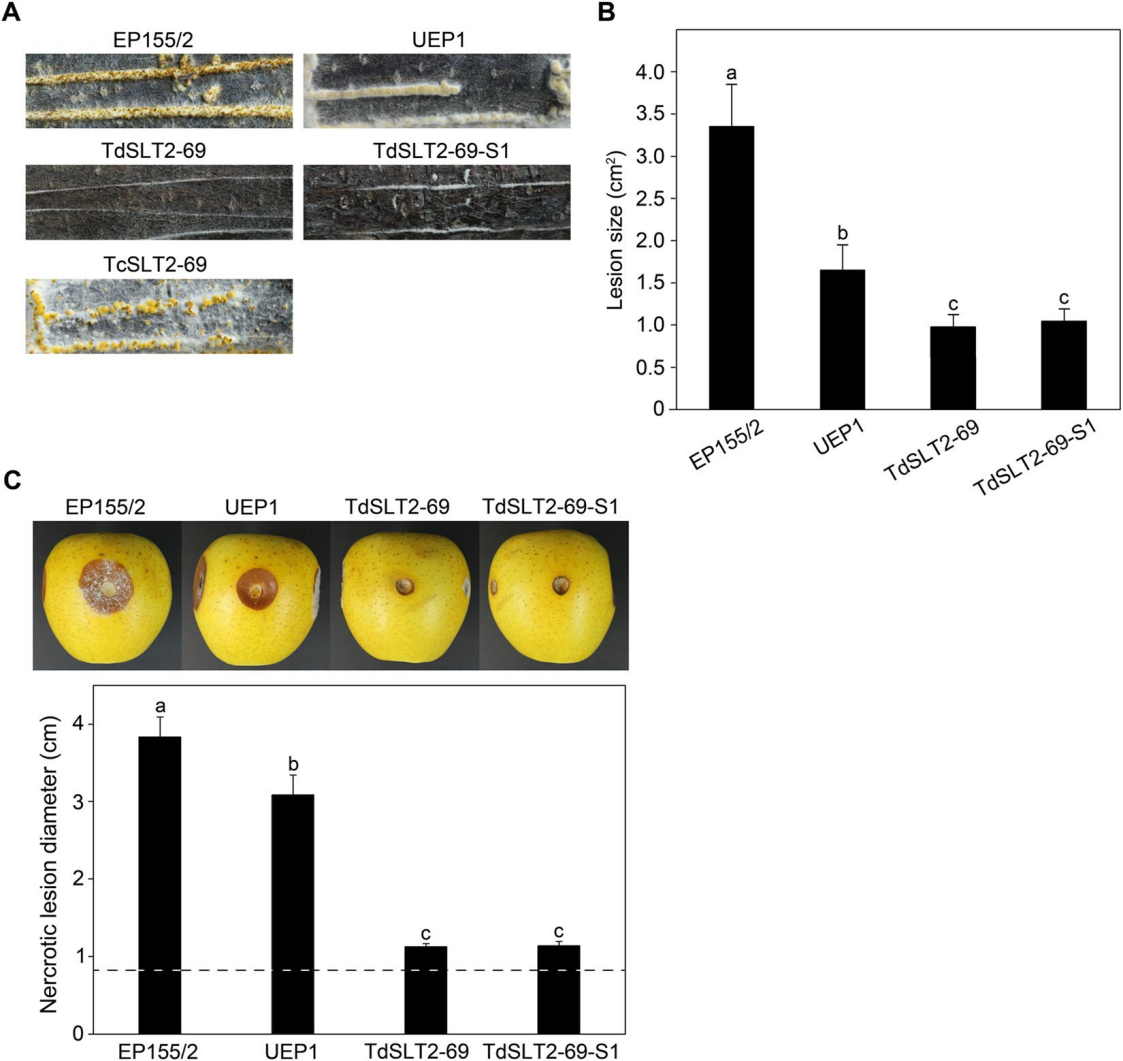
Virulence assay. (**A**) Pathogenic growth of the *CpSlt2*-null mutant strain (TdSLT2-69) on chestnut wood. Stromal pustule eruptions were observed on stem pieces of sterile chestnut wood 15 d after inoculation with wild-type EP155/2, UEP1, TdSLT2-69, the sectored TdSLT2-69 progeny, and the complemented TcSLT2-69 strains. To compare the pathogenic growth capability of the strains, the stem was artificially wounded with a razor blade and the stromal formations were compared. Note that strains with the same *CpSlt2* mutant background, such as TdSLT2-69 and TdSLT2-69-S1, showed poor stromal formation regardless of the host wound. (**B**) Virulence assay using excised tree bark as described previously^56^. Lesion measurement was conducted one week after inoculation and values are shown as the mean ± standard deviation (cm^2^). Four replicates for each strain were used, and each experiment was repeated three times. Data followed by the same letters above the bars are not significantly different between treatments, according to the Tukey HSD test at *p* = 0.01. (**C**) Apple inoculation assay as described previously^59^. Representative pictures of inoculated apples are shown 10 d after inoculation on Hwang-Ok apples and the corresponding diameters of infected discolored regions were statistically analyzed as described in (**B**). The dashed line indicates diameter induced by using 0.8 cm cork borer to punch holes on an apple for inoculation.

Since the Bavendamm assay results showed that the *CpSlt2*-null mutant greatly decreased the colored area, we next examined whether the *CpSlt2*-null mutant and its sectored progeny could grow and produce normal stroma pustules on the chestnut tree. Almost no stroma pustules were observed, indicating that the *CpSlt2* gene is very important for fungal growth and differentiation on its natural host (Fig. 7A). In addition, we inoculated the *CpSlt2*-null mutant and its sectored progeny on excised chestnut tree bark to examine whether disruption of the *CpSlt2* gene affected virulence. Consistent with results from the *CpBck1*-null mutant, the *CpSlt2*-null mutant and its sectored progeny resulted in markedly smaller necrotic areas on excised barks compared with that of the wild-type strain EP155/2. Furthermore, the necrotic area was even smaller than that of the hypovirulent UEP1 strain, indicating that the *CpSlt2*-null mutant and its sectored progeny are less virulent than both the virus-free EP155/2 wild-type strain and the hypovirulent virus-containing UEP1 strain (Fig. 7B). We then examined fungal growth and virulence using an apple assay. As shown in Fig. 7C, at most only a very small area of brown was observed around the inoculated areas by the *CpSlt2*-null mutant and its sectored progeny. Interestingly, no difference in pathogenic response was observed between the sectored progeny of the *CpSlt2*-null mutant and the parental *CpSlt2*-null mutant. This indicates that it is the CWI pathway not the growth defect that is essential for fungal pathogenic growth.

## Discussion

The MAPK pathway constitutes the signaling pathway that senses and generates the appropriate adaptive responses to changing environmental signals and other stimuli. In filamentous plant pathogenic fungi, MAPK pathways underlie the pheromone response, the CWI pathway, and the osmoregulation/stress response pathways^22^. Among these MAPK pathways, the CWI pathway of plant pathogenic fungi is primarily responsible for CWI maintenance and other species-specific processes such as virulence, sporulation, female sterility, hyphal growth and polarity, secondary metabolism, stress response, and surface hydrophobicity maintenance^37–44^. Thus, the CWI pathway is known to be significant determinant of differentiation and pathogenesis. In *C. parasitica*, *cpkk1*, an ortholog of yeast *Mkk1* in the CWI pathway, has been implicated in hypoviral regulation and fungal virulence^18, 25, 27^. Interestingly, recent studies of *CpBck1*, an ortholog of yeast *Bck1* in the CWI pathway, showed that *CpBck1*-null mutants had phenotypic changes similar to those observed in *Bck1*-null mutants of other fungi and also unique changes such as ultrastructural alterations and sporadic sectorization^26, 37^. However, studies on the molecular characterization of sporadic sectorization are very limited in filamentous fungi. This prompted us to determine whether sporadic sectorization is component-specific or a phenotypic change mediated by the CWI pathway. To do this, we examined the function of another component, *CpSlt2*, in the same CWI pathway.

Sequence analysis of the cloned *CpSlt2* gene showed the presence of a domain conserved in PKc-like superfamily members, and high homology to known fungal *Slt2* homologs. Furthermore, *CpSlt2*-null mutants exhibited cell wall-associated phenotypic changes including hypersensitivity to cell wall-disturbing agents and various other stresses. In addition, in the *CpSlt2*-null mutant, expression of the proposed downstream components of the CWI pathway, such as *Rlm1*, *Swi4*, and *Swi6*, was significantly down-regulated; in contrast, no change in the expression of the upstream component *cpkk1* was observed (Supplementary Fig. S5). Together, these results confirm that the *CpSlt2* gene encodes the MAPK of the CWI pathway. *CpSlt2*-null mutants exhibited a reduced growth rate with altered colony morphology, a near absence of conidia, and abnormal pigmentation; this suggests that the *CpSlt2* gene plays an important role in normal fungal development, consistent with its functions in other plant fungal pathogens^40–42, 45, 46^ (Supplementary Fig. S6). Moreover, as was the case for the *CpBck1*-null mutant, the *CpSlt2*-null mutant exhibited sporadic sectorization and, once sectored, robust mycelial growth without differentiation was stably maintained during successive transfers of sectored progeny. Complementation of the sectored progeny with the wild-type allele of *CpSlt2* confirmed that disruption of the *CpSlt2* gene resulted in the sectored phenotype. These results show that sporadic sectorization of *C. parasitica* is related to the CWI pathway. This is a novel phenotypic change, which has not been described as a known CWI defect in other fungi. In *C. parasitica*, sectorization has never been observed in a HOG1 mutant^16^ or a mutant of the *cpmk2* gene, an ortholog of yeast *Fus3* ^23^. Thus, sectorization appears to be a CWI-specific phenotypic change (Supplementary Fig. S6). Based on the characteristics of sectorization such as the several days required for its development, sectorization of only restricted mycelial regions, maintenance of stable inheritance of the sectored phenotype (once sectored), and no changes in genome integrity, epigenetic studies focusing on the explanation of the genetic nature of sectorization are underway^47^. Moreover, our results indicate that the sectored progeny of mutants had dramatic genome-wide changes in pre-existing DNA methylation, a prototypical epigenetic process (manuscript in submission).

Although *Mkk1* mutants were not included in this study, the phenotypic changes and responses to the majority of the tested conditions clearly showed that *CpSlt2* and *CpBck1* play consorted roles in CWI, fungal growth, morphogenesis, responses to external stimuli responses, host infection, and sectorization. However, *CpSlt2*-null and *CpBck1*-null mutants exhibited differences in apical hyphal growth units. Besides difference in the apical hyphal growth units, the *CpSlt2*-null mutant showed the same or less severe abnormalities, and less sensitivity to stress compared with the *CpBck1*-null mutant, indicating that the mutant did have differences. Therefore, it is possible that components even in the same CWI pathway may have its own, not-yet-identified, functions. In addition, crosstalk with other MAPK pathways was involved in the physiological response to a specific activating signal. Sequential activation of the HOG1 and CWI pathways has been identified in *S. cerevisiae* ^48^. Considering the increased osmosensitivity of the *CpSlt2*-null mutant, it is conceivable that functional crosstalk between the CWI and HOG1 pathways occurs in this fungus. Therefore, crosstalk with other unknown pathways may further increase the complexity of component- and pathway-specific responses. Further studies on two similar, but not identical, mutants will allow us to define the pathway- and component-specific target genes, which will help us understand the global regulatory roles of the CWI pathway.

## Methods

### Fungal strains and growth

The wild-type *C. parasitica* strain EP155/2 (ATCC 38755) and its isogenic CHV1-713-infected strain UEP1 were maintained on a plate containing potato dextrose agar (PDA) supplemented with L-methionine (0.1 g/L) and biotin (1 mg/L) at 25 °C with constant low light^49^. The fungal culture conditions and methods for the preparation of the primary inoculum for the liquid cultures are previously described^16, 49^. The harvested mycelia were stored at −70 °C and lyophilized for stability as described previously^50^.

### Cloning and characterization of *CpSlt2*, a cell wall integrity MAPK gene

The genome database of *C. parasitica* was screened for the *Slt2* homolog and PCR amplification was performed using the primers *CpSlt2*-gF1 (forward) 5′-CTAAACCAACTGTCGGATGC-3′ and *CpSlt2*-gR1 (reverse) 5′-CCAGCACAGGGTGTAAGT-3′. The resulting 6.8-kb PCR amplicon was cloned into a pGEM-T Easy vector (Promega, Madison, WI, U.S.A.) and sequenced using the dideoxynucleotide method with universal and synthetic oligonucleotide primers.

To obtain the cDNA clone of *CpSlt2*, PCR using reverse transcriptase (RT-PCR) was performed with primers *CpSlt2*-cF1 (forward) 5′-CAATTCAAGATGGCAGAGTTA-3′ and *CpSlt2*-cR1 (reverse) 5′-GTGAGACTCTATCTCCTCCCG-3′ at nucleotide (nt) positions −9 to 12 and 1,644 to 1,664 (relative to the start codon), respectively. The resulting 1.3-kb cDNA amplicon was cloned and sequenced.

### Southern blot analysis

Genomic DNA from *C. parasitica* was extracted as described previously (Churchill *et al*.)^52^. The genomic DNA (20 μg) was digested with the restriction enzyme *Sac*II, blotted onto nylon membrane, and hybridized with radioactive-labeled probes.

### Construction of a replacement vector and fungal transformation

The replacement vector pDSLT2, which was designed to favor double-crossover integration events, was constructed as follows: The pDSLT2 vector consists of three parts, the 5′- and 3′-flanking regions of the *CpSlt2* gene, and the hygromycin B phosphotransferase (*hph*) gene cassette. A 6.8-kb fragment containing full-length *CpSlt2* was ligated into a pGEM-T Easy Vector, and the resulting plasmid was used as the template to obtain the 5′- and 3′- flanking regions of the *CpSlt2* gene. The PCR amplicons of the 5′- and 3′-flanking regions were amplified with primers *CpSlt2*–5′FL-F1 (forward) 5′-TCTACCATGAGGCCGTTCGA-3′, *CpSlt2*-5′FL-R1 (reverse) 5′-TCCTTCAATATCATCTTCTGTCGACACCGAGGACAGGGTTCTGGA-3′, *CpSlt2*-3′FL-F1 (forward) 5′-GCATAAGGGAGAGCGTCGACTGCCGACGGGAGGAGATAGA-3′, and *CpSlt2*-3′FL-R1 (reverse) 5′-GCACGTTCATCCAAGCCCTG-3′ to incorporate the *hph* site for fusion PCR (underlined). The *hph* cassette was amplified with primers hph-F1 (forward) 5′-TCCAGAACCCTGTCCTCGGTGTCGACAGAAGATGATATTGAAGGA-3′ and hph-R1 (reverse) 5′-TCTATCTCCTCCCGTCGGCAGTCGACGCTCTCCCTTATGC-3′ to incorporate the 5′- and 3′-flanking regions of *CpSlt2* for fusion PCR (underlined). Furthermore, three PCR amplicons were overlapped by fusion PCR with primers *CpSlt2*-5′FL-F1 and *CpSlt2*-3′FL-R1. The resulting 5.1-kb PCR amplicon was cloned into the pGEM-T Easy vector and sequenced. In the replacement vector pDSLT2, the *hph* cassette was inserted between −37 and 1,637 bp of the *CpSlt2* gene relative to the start codon and was flanked by approximately 1.3 and 1.4 kb of the 5′ and 3′ sequences, respectively. The 5.1-kb *Not*I fragment of pDSLT2 was then used to transform the virus-free EP155/2 strain.

Functional complementation of the *CpSlt2*-null mutant was performed using the wild-type allele. The complementing vector pCSLT2 was constructed by inserting a *Not*I*/Xba*I-digested 5.5-kb fragment containing the full-length *CpSlt2* gene into a *Not*I*/Xba*I-digested pSilent-Dual1G (pSD1G) vector containing the geneticin resistance cassette^51^. The resulting vector was then used to transform the *CpSlt2*-null mutant.

Protoplast preparation and transformation of *C. parasitica* were performed as described previously^15, 16, 52^. Transformants were selected from PDAmb plates supplemented with 150 μg/mL hygromycin B (Calbiochem, San Diego, CA, U.S.A.) or 150 μg/mL geneticin (Invitrogen, Carlsbad, CA, U.S.A.), passaged three to four times on selective medium, and single-spore isolated, as described previously^15, 53^. PCR and Southern blot analysis were conducted with genomic DNA from the transformants to confirm the replacement and *in trans* complementation of the *CpSlt2* gene.

### Characteristics of the *CpSlt2*-null mutant

The phenotypic and molecular characteristics of the *CpSlt2*-null mutant were compared with wild-type EP155/2 and its isogenic hypovirulent UEP1 strains. Phenotypic changes in growth rate, pigmentation, conidiation, and mating capability were measured using previously described methods^15, 50^. Morphological characteristics were investigated on PDAmb medium with cell wall perturbing agents such as Congo red (CR), and sodium dodecyl sulfate (SDS)^54^. To examine the osmosensitivity, the *CpSlt2*-null mutant was transferred to PDAmb supplemented with four different concentrations (0.25, 0.5, 1.0, 2.0, and 2.5 M) of sorbitol^16^. Responses to ROS stress were examined on menadione-containing PDAmb, which causes odidative stress^55^. A virulence test using a piece of chestnut tree bark was conducted according to Lee *et al*.^56^.

### Microscopic observation

Fungal mycelia were cultured for 5 d on PDAmb and viewed under differential interference contrast (DIC) microscopy (BX53, Olympus) to analyze the blanching pattern of *C. parasitica* hyphae. To quantify the changes in the branching pattern, ProRes^®^ CapturePro software (version 2.8.8, JENOPTIK) was used to measure the straight length distance from an apical tip to a third-order branch of hyphae.

To evaluate the CWI of hyphae of *C. parasitica* strains, fungal mycelia were cultured for 10 d on PDAmb and then analyzed by SEM and TEM. Briefly, mycelial fragments detached from the outermost region of fungal colonies were fixed with modified Karnovsky’s fixative in 0.05 M sodium cacodylate buffer supplemented with 2% glutaraldehyde and 2% paraformaldehyde at pH 7.2^57^. After post-fixation with 1% osmium tetroxide in 0.05 M sodium cacodylate buffer at pH 7.2 and dehydration through a graded ethanol series of 30, 40, 50, 70, 80, 90, and 100% for 10 min each, the specimens were coated with gold using a Sputter Coater (JFC-1100E, JEOL, Tokyo, Japan). The specimens were observed with a scanning electron microscope (SN-3000, Hitachi, Japan) at 20 kV^31^.

After dehydration of the mycelia, the fungal samples were embedded in EPON 812 resin following the standard procedures for specimen preparation for TEM^31, 58^. For light microscopy, semi-thin (0.5- to 1-μm) sections were made with a diamond knife after staining with 1% toluidine blue O. Ultra-thin (70- to 80-nm) sections of embedded samples were cut with a diamond knife, stained with 2% uranyl acetate and Reynold’s lead citrate, and observed using a transmission electron microscope (H-7650, Hitachi, Japan) at 100 kV.

### Statistical analysis

Colony diameters and canker areas were analyzed by ANOVA using SPSS software (version 23.0, SPSS Inc.) and the significance of the effects of the strains and treatments was determined using the Tukey honest significant difference (HSD) test at *p* = 0.01.

## Electronic supplementary material

Supplementary information
