# Supplementary material for: Mutation of the Slt2 ortholog from Cryphonectria parasitica results in abnormal cell wall integrity and sectorization with impaired pathogenicity
Source: Sci Rep. 2017 Aug 22;7:9038. doi: 10.1038/s41598-017-09383-y (PMC5567307; doi:10.1038/s41598-017-09383-y)
Supplement: Supplementary file 1 — Supplementary information [file 41598_2017_9383_MOESM1_ESM.pdf]

Supplementary Information

Mutation of the *Slt2* ortholog from *Cryphonectria parasitica* results in abnormal cell wall integrity and sectorization with impaired pathogenicity

Kum-Kang So[1], Yo-Han Ko[1], Jeesun Chun[1], Jung-Mi Kim[2] & Dae-Hyuk Kim[1*]

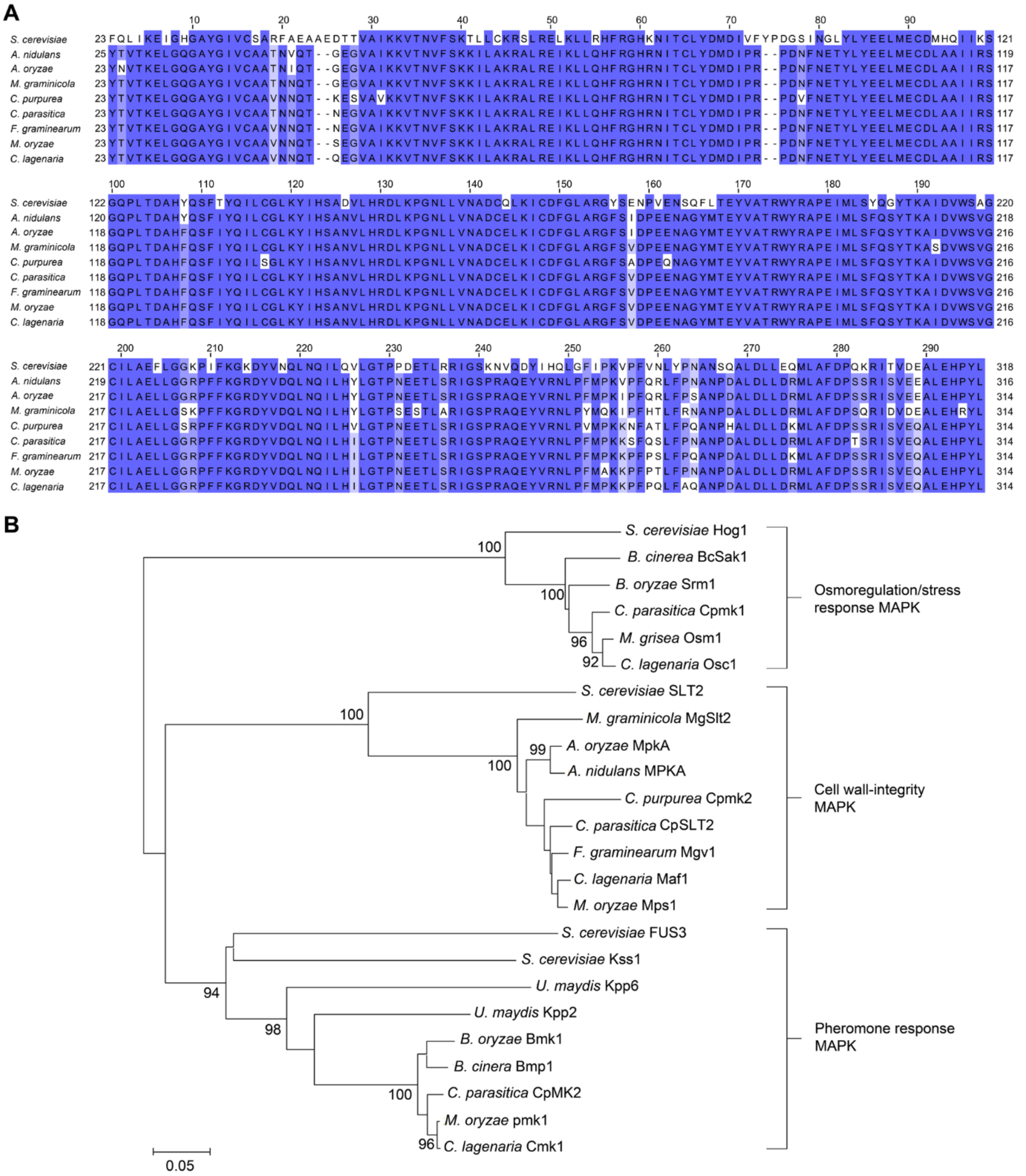

Figure S1. Multiple alignment and phylogenetic analysis indicate that the cloned *CpSlt2* gene encodes a Slt2-related MAPK. (**A**) Alignment of amino acid sequences of the conserved PKc-like superfamily domain of the predicted *CpSlt2* gene product with known *Slt2* homologs from the following fungi: *Aspergillus nidulans* (GenBank No. AAD24428), *A. oryzae* (GenBank No. XP_001816695), *Claviceps purpurea* (GenBank No. CAC87145),

Colletotrichum lagenarium (GenBank No. AAL50116), *Fusarium graminearum* (GenBank No. XP_003712437), *Magnaporthe oryzae* (GenBank No. XP_003712437), and *Mycosphaerella graminicol* (GenBank No. AAY98511). Identical amino acids are highlighted in dark grey and similar amino acids are shaded. Dashes represent gaps in the alignments. The residue number from the N-terminus of the whole protein is shown at left and right. (**B**) Phylogenetic comparison of closely related *Slt2*s with other characterized fungal groups of MAPKs. The MAPKs representing the pheromone response pathway were from *Botrytis cinera* (GenBank No. AAG23132), *Bipolaris oryzae* (GenBank No. BAD42855), *C. lagenarium* (GenBank No. AAD50496), *C. parasitica* (GenBank No. AAP86959), *M. oryzae* (GenBank No. AAC49521), *S. cerevisiae* (GenBank No. AAZ22456), *S. cerevisiae* (GenBank No. CAA84835), *U. maydis* (GenBank No. AAF15528), and *U. maydis* (GenBank No. CAD43731). MAPKs representing the osmoregulation/stress response pathway were from *Botrytis cinera* (GenBank No. AHL24869), *Bipolaris oryzae* (GenBank No. BAE48722), *C. lagenarium* (GenBank No. BAD11137), *C. parasitica* (GenBank No. AAO27796), *M. oryzae* (GenBank No. AAF09475), and *S. cerevisiae* (GenBank No. CAA97680). An evolutionary matrix was generated using the Henikoff & Henikoff (1992) method[1], and the branching pattern was generated using the neighbor-joining method. The numbers at the nodes indicate bootstrap values and a scale for branch length of dissimilarity is indicated at the bottom.

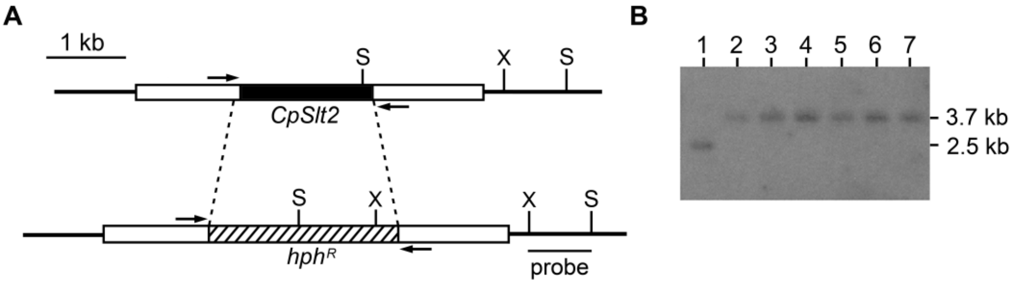

Figure S2. Southern blot analysis of the *CpSlt2*-null mutants (TdSLT2-42 and -69). (**A**) Restriction map of the *CpSlt2* genomic region and the site of the expected replaced allele of the *CpSlt2* gene due to double homologous recombination. Boxes indicate the genomic region for the construction of the gene replacement vector pDSLT2, which replaced the internal 1,932 bp with the hygromycin B resistance gene (*hph*), resulting in 1.3- and 1.4-kb fragments as the 5'- and 3'-flanking regions, respectively. S and X indicate the restriction site of *Sac*II and *Xba*I, respectively. Primers used to confirm gene replacement at positions (nt) -128 to -109 and 1,717 to 1,736 (relative to the start codon of *CpSlt2*) are indicated by arrows. (**B**) Southern blot analysis of *Sac*II-digested DNA from the wild-type EP155/2 strain (lane 1) and three single-spored clones of TdSLT2-42 (lanes 2-4) and TdSLT2-69 (lanes 5-7) transformants. The probe prepared from a 0.9-kb *Xba*I/*Sac*II-digested *CpSlt2* fragment is shown on the restriction map in the panel A and the 1.0-kb size bar is shown on the top left. Note that the transformants TdSLT2-42 and -69 had undergone the desired replacement at *CpSlt2*, as evidenced by the expected size change of the hybridizing band.

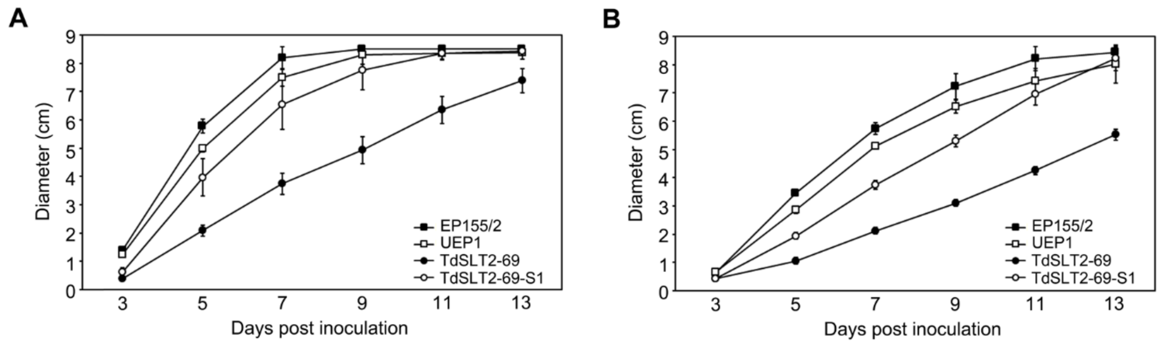

Figure S3. Mycelial growth rate of the *CpSlt2*-null mutant strain. Diameters of colonial growth were measured every day at 25 °C (**A**) and 20 °C (**B**) for 13 d. Six replicates for each strain were used, and each experiment was repeated twice. Compared with the wild type, no significant changes due to temperature were observed.

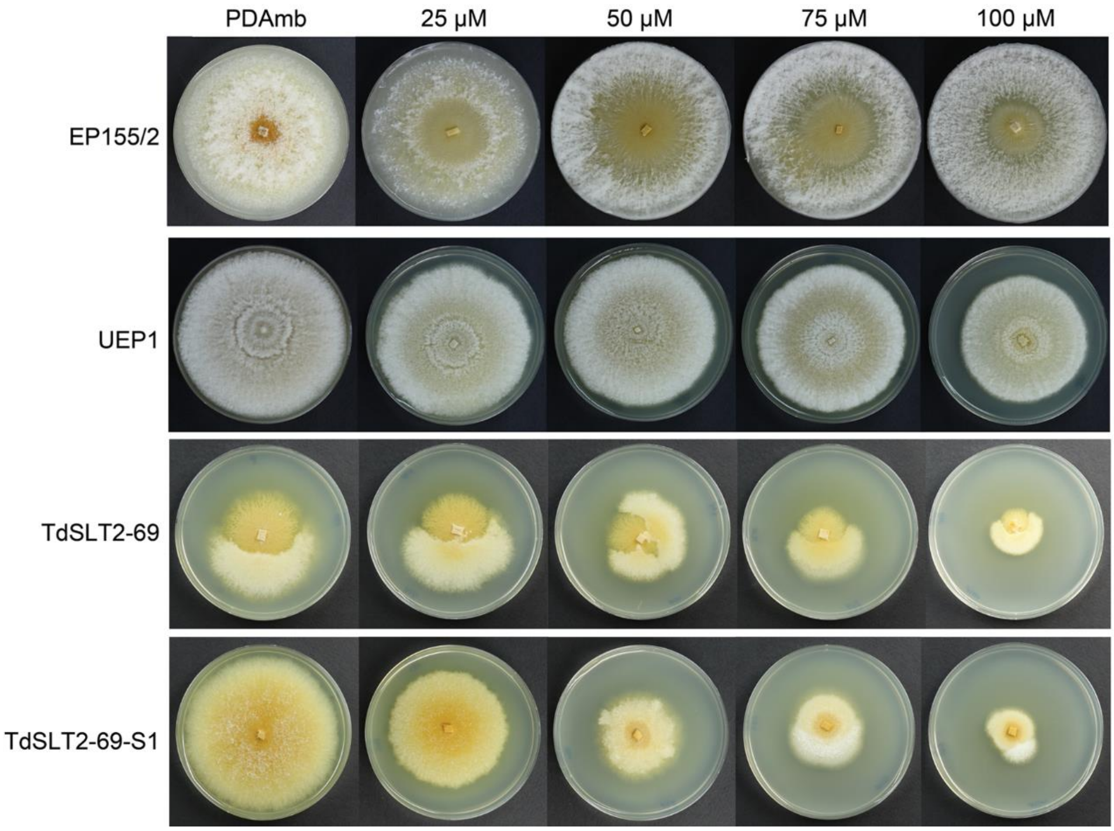

Figure S4. Effect of ROS on fungal growth. Colony morphology on PDAmb supplemented with corresponding concentrations of menadione after 10 d of cultivation. Strains shown are wild-type EP155/2, UEP1, TdSLT2-69, and the sectored TdSLT2-69 progeny, TdSLT2-69-S1. Note that colonies from the same *CpSlt2* mutant background, such as TdSLT2-69 and TdSLT2-69-S1, showed severe growth defects in a dose-dependent manner when grown in 100 μM of menadione. However, menadione did not significantly affect the fungal growth of wild type and its isogenic hypovirulent UEP1 strain, compared with grown on PDAmb only.

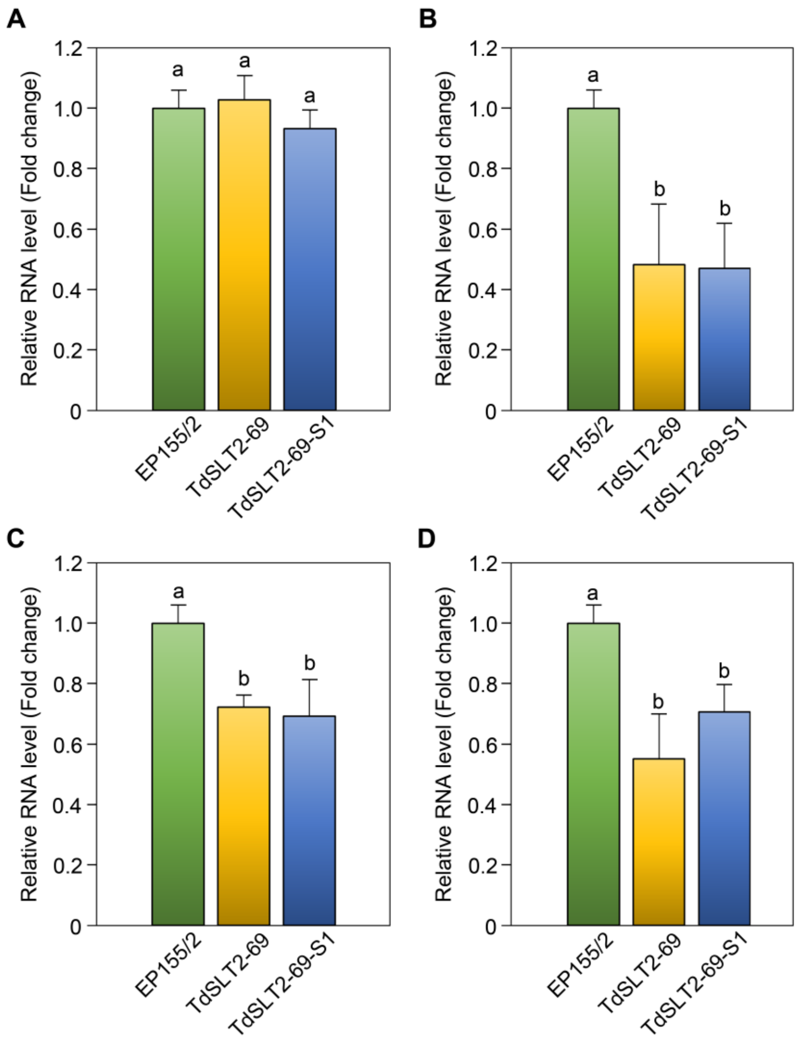

Figure S5. Molecular phenotype of the *CpSlt2*-null mutant. Expression of up- and downstream components of the proposed CWI pathway in the *CpSlt2*-null mutant compared with the wild type. Quantitative real-time RT-PCR analysis of the transcript levels of *cpkk1* (**A**) and its yeast orthologs *Rlm1* (**B**), *Swi4* (**C**), and *Swi6* (**D**) relative to that of β-tubulin (GenBank No. GU993733) as described previously[2]. Values on the *y*-axis were normalized to the transcript level of the corresponding gene in the EP155/2 strain after culture for 5 days; error bars indicated standard deviations of three independent measurements. The wild-type EP155/2, TdSLT2-69, and the sectored TdSLT2-69 progeny TdSLT2-69-S1 are shown. *In silico* analyses of a genome database (http://genome.jgi-psf.org/Crypa1/Crypa1.home.html)

using the corresponding yeast genes revealed that Crypa1.estExt_Genewise1.C_41737, fgenesh1_pm.9_#_5, and fgenesh1_pm.3_#_49 are orthologs of *Rlm1* (**B**), *Swi4* (**C**), and *Swi6* (**D**), respectively.

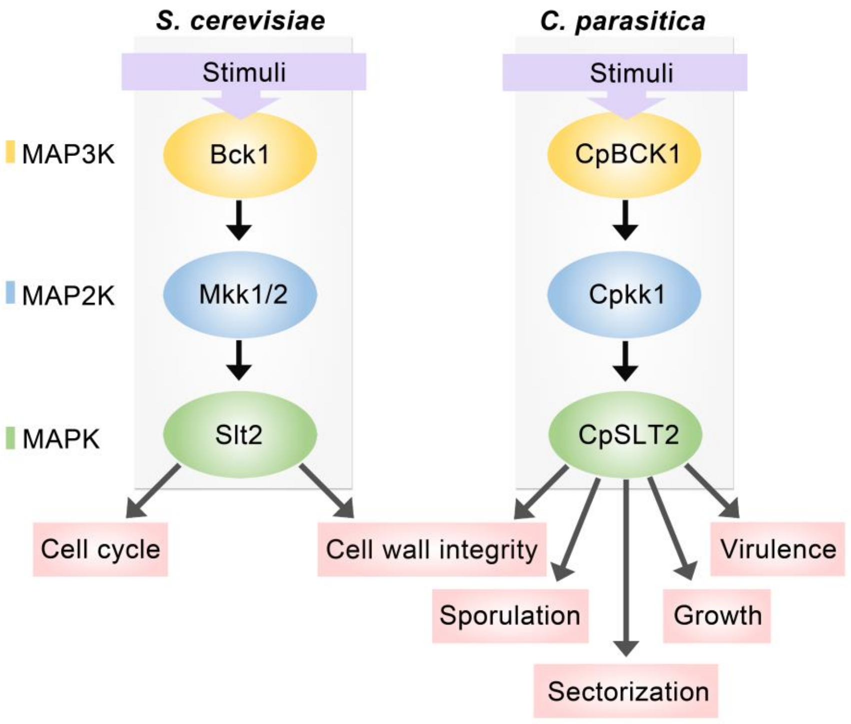

Figure S6. Comparative schematic diagram of the CWI pathway in *C. parasitica*. Ovals represent genes, and rectangles represent phenotypes.

Supplementary Reference

1. Henikoff, S. & Henikoff, J. G. Amino acid substitution matrices from protein blocks. *Proc. Natl. Acad. Sci. U. S. A.* **89,** 10915-10919 (1992).
2. Kim, M. J., Choi, J. W., Park, S. M., Cha, B. J., Yang, M. S. & Kim, D. H. Characterization of a fungal protein kinase from *Cryphonectria parasitica* and its transcriptional upregulation by hypovirus. *Mol. Microbiol.* **45,** 933-941 (2002).
